# The role of school desk on the learning of graphic skills in early childhood education in Brazil

**DOI:** 10.1186/s40064-016-2554-1

**Published:** 2016-07-19

**Authors:** Roberto Gimenez, Rafael do Nascimento Soares, Victor Vedovelli Ojeda, Cristiane Makida-Dionísio, Edison de J. Manoel

**Affiliations:** University of the São Paulo City (UNICID), Rua Cesário Galero, 448/475, São Paulo, SP 03071-000 Brazil; Department of Pedagogy of Human Movement, School of Physical Education and Sport, University of São Paulo (USP), Av. Prof. Melo Morais, 65, São Paulo, SP 05508-030 Brazil

**Keywords:** School desk, Graphic skill, Skill acquisition, Early childhood education

## Abstract

The role of two different layouts of school furniture was investigated in the pattern legibility and spatial–temporal parameters of a graphic skill acquisition. Thirty children from the first grade of elementary school (mean age = 6 years) practiced a graphic task according to a criterion figure. They were assigned to two groups, Group of Fixed School Desk (GF) and Group with Adjustable School Desk (GA). Each child practiced the task on a digital tablet for 25 trials. The software *Movalyser* 2.3 processed the data from which the following measures were obtained: pattern legibility, linear spatial error and speed of execution. Two expert teachers also judged legibility. Children in the GA showed more number of legible patterns, they were slower to complete the task but they were more accurate in its reproduction. The adjustable school desk facilitates the acquisition of legible graphic patterns. Since stable graphic skills are positively correlated to the production of creative texts, studies unraveling the role of school desks to facilitate handwriting and drawing skills will contribute ultimately children’s literacy and overall educational development.

## Background

Learning to write has been a key process in Education as it is vital for literacy. Postman (1994) argues that childhood was “invented” when the need to become literate was urgent in the eighteenth century Europe. Since then, graphic and handwriting skills have been cherished as truly motor milestones in childhood alongside walking, speaking and tool use. Even though in the twenty-first century the use of notebooks and tablets with their keyboard and touch screen, respectively, is increasing among children, there is evidence that handwriting is a skill that needs to be acquired prior to keyboarding skills (Stevenson and Just 2014). Learning to write entails the ability to transcribe sounds in a coded system with a particular grammar necessary to communicate properly with a potential reader (Briggs 1970). In this sense, the ability to communicate by means of a drawing is intertwined with the ability to write. For instance, Bottrell (2011) postulates that “skills for making graphic marks are interchangeable with drawing skills…writing and drawing involve skills for making configurations resulting in symbols that carry meaning” (p. 308).

The motor skill to write and drawn is critical during the school period as it is involved almost in all school activities. Christensen (2004) points out a significant correlation between the automaticity to perform graphic skills and the production of creative quality texts. The difficulties to perform writing and drawing patterns have been also associated with learning problems in general (e.g. Berninger et al. 1997; Jones and Christensen 1999), and for young learners, the problems are also related to letter formation and reading (Graham and Harris 2005; Vander Hart et al. 2010). Bearing in mind that children with handwriting difficulties are prone to have their ability to learn minimized in various dimensions (Coates and Coates 2006), the understanding of how children acquire handwriting skills is of a practical value for school teaching. For instance, Bara and Gentaz (2013) designed two instruction programs for handwriting, one based on visual inspection of letters and another involving visual as well as haptic exploration of letters. After five sessions they found that 5 year-old children who took part in the visual-haptic instruction program perform better in handwriting copying tasks than those children who experienced only visual inspection.

The study of handwriting and drawing skills has been a field on its own known under the term graphonomics that aims to investigate the planning and organization of theses actions and its relationship with the resulting spatial traces (International Graphonomics Society 2015). It has been shown that handwriting and drawing skills entail the formation of action programs with a memory representation of the sequencing and timing of strokes (Gimenez et al. 2006; Manoel et al. 2002). One interesting finding with educational implications was that the acquisition of handwriting and drawing skills involve a crescent incorporation of simple programs into others with more complexity (Kharraz-Tavakol et al. 2002). This process called modularization by Connolly and Bruner (1974) has been found to occur in the acquisition of graphic skills in children (Manoel et al. 2011). In the practice of basic strokes that form letters and figures, children will acquire module, simple memory representations that will be taken to form complex action programs which allow them to words and sentences with less attention in the mechanics of the action leaving mental space for the symbolic processing necessary for all the semantic memory.

Cahill (2009) has called attention for the multiplicity of factors involved in the performance and acquisition of graphic skills. The studies have looked at (a) the paper position in regard to the subject (Edwards 2003); (b) type of paper, rule or plain (Daly et al. 2003; Fitzpatrick et al. 2013); (c) the distance between lines in the ruled paper (Graham 1992); (d) the use pencil adjusted to beginners (Ascher 2006). The lay out of furniture is thought also to influence the performance and acquisition to children’s handwriting and drawing skills (Feder and Majnemer 2007). The lay out of school desks has been the subject of ergonomics with studies focusing on postural control and overall motor performance in handwriting (e.g. Green and Nelham 1991; Nowak 1996; Dean et al. 1999). Children should be encouraged to sit in school chairs with their hips, knees and ankles at 90°, their feet fully supported on the floor and with their arms being supported comfortably on the table that need to be slightly inclined (c.f. Amudson 2005). The adequate child-school desk interaction requires that the second be adjustable to the child’s height. However, the effectivity of school furniture has not been studied let alone adjustable school desks. There are some studies on the impact of the layout of furniture on the handwriting of cerebral palsy children (Hadders-Algra et al. 2007). There is one study that verified the impact of two different layouts of school desks on printing legibility of young primary grade students with cerebral palsy (Stephen et al. 2010). The study failed to find evidence that adapted and specially designed school desk had a positive impact on the children’s print legibility. Similar studies corroborated the results of this study (Bracialli et al. 2008; Bracialli and Villarta 2000; Shen et al. 2003).

There is indeed a gap in the understanding of the impact of school furniture’s layout in the acquisition of graphic skills of typically developing children. The gap is wider if one considers that public policies worldwide paid little attention to the impact school desk can have on children’s handwriting and indeed on all literacy (Domljan et al. 2008). In Brazil, there are few schools with furniture adjustable to the children’s anthropometric dimensions. In a survey conducted in the State of São Paulo (one of the richest states in Brazil), it was found that the vast majority of public schools neglects the role of school desk on children’s learning. In particular, Early Childhood Education is a school period when studies should concentrate, as it is the time for the acquisition of handwriting and drawing skills. The practice of these skills occurs in different types of furniture and is not sufficiently clear the impact their lay out have on the acquisition of graphic skills. Apart from the lack of studies on the impact of school desk, the literature on the ergonomics of furniture has looked on the impact different lay out have on overall motor performance, e.g. letter legibility. Whereas good writing can be understood as the ability to convey a message through graphic records, it is considered that legibility could indicate the skillful writing. Good writers will also have good letter legibility which means being capable of reproducing graphic patterns with relative precision and accuracy, and speed of execution (Graham et al. 2006).

From the literature review, we can sum that the investigation of the impact of furniture on children’s handwriting and drawing should consider two main variables: (1) to investigate different layouts of school desks, as an independent variable; (2) describe the effects on pattern legibility, spatial and temporal aspects of children’ strokes, these being dependent variables. These last three variables are related to the memory representation that is formed during the handwriting and drawing experiences. Pattern legibility is related to the sequence of action and the spatial and temporal parameters of the strokes are related to relative timing and force (Manoel et al. 2002; Gimenez et al. 2006). The memory representation of graphic skills is likely to be part of a larger competence involved in literacy. School desks that favors pattern legibility and the stability of spatial and temporal patterns of strokes may have a positive impact on children’s literacy. The goal of the present study was to investigate the effect of two layouts of school desks on pattern legibility and spatial and temporal parameters of graphic skills practiced by typically developing children attending elementary school.

## Methods

### Participants

Thirty children took part in the study. The children’s parents were briefed about the research goal and procedure and agreed with the participation of their children. Parents signed an informed consent form in accord with the requirements of the Ethics Committee for Research of the University of Sao Paulo, Brazil. All children came from two elementary schools and were eligible to take part in the study once from school records they were considered typically developing children. Exclusion criteria included children who had any developmental disorder such as attention-deficit hyperactivity, autism spectrum disorder or any classical neurological sign such cerebral palsy. The children were randomly assigned to one of two groups defined by the school furniture layout: Group Fixed School Desk (GF) with seven boys and eight girls (n = 15), mean age of 6 years and 9 months, and Group Adjustable School Desk (GA) with nine boys and six girls (n = 15), mean age of 6 years and 7 months.

### Experimental task and apparatus

**Fig. 1:**
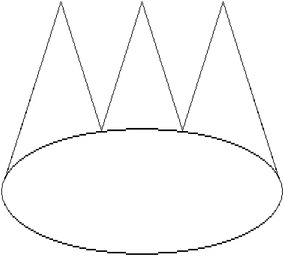
Criterion model figure the king ‘s crown

The task involved the reproduction of a graphic pattern similar to a king’s crown (Fig. 1). This graphic pattern was defined after a pilot study and for having two advantages: (a) it was easily recognizable by children who associated it with a kind of a crown; (b) it involved a combination of traces with linear and semicircular shapes that made the task demanding in two dimensions: (a) The spatio-temporal pattern to integrate strokes; (b) The sequence of strokes. The graphic pattern was tested in a pilot study and proved to be easily recognised by children with similar ages.

The reproduction of the graphic pattern was made ​​on a AIPTEC Tablet, model 8000 U, with a cordless sensitive pressure pen. The tablet was connected to a notebook model Hewlett-Packard Pavillion Intel Dual Core. The collected data was treated by a Software *MovAlyzer*, version 3.2, developed by Neuroscript Group. This software recognized children’s strokes in terms of space and time allowing for the description of the graphic produced in terms of stroke speed, timing and sequence.

### Procedure

**Fig. 2:**
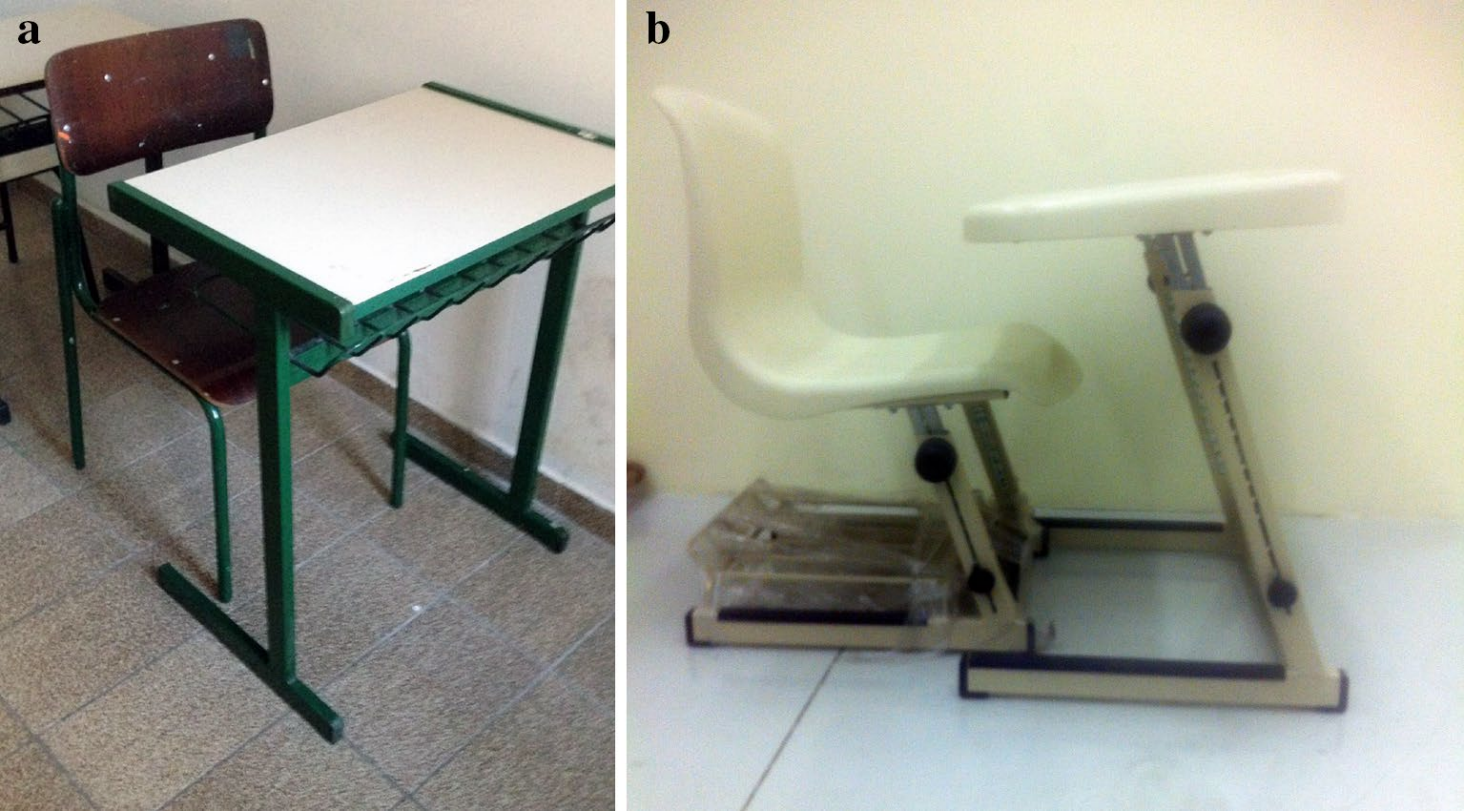
School desks: **a** fixed and **b** adjustable

The study focused on the effect of two kinds of school furniture, one standard and fixed (GF), and another that could to be adjustable (GA) to the child’s physical dimensions (Fig. 2). Children in the GF practiced the graphic pattern in a standard school desk commonly used in most public schools in São Paulo, Brazil. In the other group, GA, children sat in a school furniture in which the height of the chair and the desk could be adjustable to the children’s anthropometrical dimensions. The school desk was adjusted to each child in order for he or she to sit with their hips, knees and ankles at 90°, their feet fully supported on the floor and with their arms being supported comfortably on the table that was slightly inclined. The children’s height was controlled by the use of the individual’s mean height in the sample. The children who exceeded the mean height of 116 cm in more than 6 centimetres were not considered for data analysis.

The study was conducted in a quiet room in the elementary school attended by the children studied, following an authorization given by the School Principal. In accord with the classroom teacher, each child was invited to take part in a graphic task that was going to take place in another room. Once there, the child was asked whether he or she recognized the figure and then whether he or she could drawn it. The child was asked to reproduce the pattern as accurate and fast as possible in 25 trials. The criterion figure was presented at the top of each sheet serving as model to the children. The instructions stated that the king’s crown should present three tips and a rounded base. The experimenter asked the child to perform the task with his or her preferred hand and not the change hands during practice. The child had visual feedback about the spatial features of the patterns produced and also the time taken to perform it.

### Measures and hypothesis

From the record of strokes the quality of the graphic skill was assessed by two evaluators who judged the degree of legibility considered from the correspondence between the model and the actual drawing. Graphic reproductions were considered legible when the three tips and the rounded base of the crown could be identified. The agreement between the evaluators judgments was tested by an inter-rater agreement index proposed by Thomas et al. (2011). The values ​​obtained were higher than 0.81 which is considered a very satisfactory agreement. Another qualitative measurement was the posture adopted by the children in each kind of school desk. The categorization was based on four body posture components thought to be adequate to optimize handwriting performance: support on the chair backrest; elbow support in the desk; hand support on the paper and support of the feet.

Legibility was further used as a control to eliminate from the quantitative analysis the drawings that did not correspond to the model. The software *MovAlyzer* made a recognition analysis of all patterns drawn automatically discarding those that did not match the specifications. The quantitative analysis was based on two measures: total movement time and spatial linear error. Total Movement Time comprehended the time interval from the start of the first stroke to the conclusion of the drawing calculated in seconds. Spatial linear error was calculated as the linear size difference in centimetres between criterion figure (the model) and the actual drawing performed by the children.

The main assumption made in the present study was that the lay out of school desk will interfere with the legibility of the drawing, body posture and also with the spatial and temporal aspects of graphic patterns. Hence, we hypothesized that adjustable school desk will provide better conditions for practicing the drawing. This benefit will be manifested in the children’s performance with the GA doing better than GF in every account.

### Statistical analysis

For the purpose of the descriptive and inferential statistics, the 25 trials of the practice session were grouped in five blocks with five trials each. The Univariate Cochran’s C and Brown-Forsythe tests were used to test for normality of the resulting data. The differences in pattern legibility and spatio-temporal parameters between the two layouts of school furniture during the practice of the task were tested by a Two-Way ANOVA, Group (2) × Blocks (5) with repeated measures in the second factor. Whenever a significant *F*-ratio was obtained, the Tukey post hoc test with Bonferroni correction was used to locate the differences. For all statistical analyses, significance was accepted at *p* < 0.05. The Contingency Correlation test was also calculated to search for significant associations between body postures components and legibility of patterns. This type of test is recommended to verify the existence of associations between nominal variables. The statistical analysis was performed using Statistical Package for Social Sciences (SPSS, version 22.0).

## Results

We expected that adjustable school furniture would allow a posture more appropriate to handwriting and drawing. Indeed, we did find that children from the GA had their body posture’s components organized to favour postures more adequate to handwriting and drawing (Table 1). The body posture’s components surveyed were (a) Back: with back support and without back support; (b) Elbow: total elbow support, partial elbow support, without elbow support; (c) Hand: total hand support; partial hand support; without hand support; (d) Feet: total feet support, partial feet support and without feet support. Drawing without back support was predominant in both groups; still 33 % of the children form the GA rest their back on the chair while only 7 % of the children in the GF did the same. The fixed school furniture (GF) led more children to show total elbow support (67 %). In this same component, Children in the adjustable school furniture did not show any preference with 47 % of the children with total elbow support and 40 % without elbow support. A similar pattern was observed with the Hand component. The majority of children in the GF showed total hand support (67 %), while in the GA children were nearly equally distributed with 46.5 % with total hand support and 46.5 % without hand support. The most noticeable trend was for the Feet component, with adjustable school desk (GA) favouring an adequate posture by keeping feet in total (33 %) or partial (60 %) contact with the floor. In the other group, GF, it was quite the opposite with 60 % of the children without feet support and only 7 % of them with total feet support.

**Table 1:** Survey of body posture’s components during practice

Components	Fixed school desk [GF (%)]	Adjustable school desk [GA (%)]
Back		
BS	7	33
WBS	93	67
Elbow		
TES	67	47
PES	20	13
WES	13	40
Hand		
THS	67	46.5
PHS	7	7
WHS	26	46.5
Foot		
TFS	7	33
PFS	33	60
WFS	60	7

*BS* back support, *WBS* without back support, *TES* total elbow support, *PES* partial elbow support, *WES* without elbow support, *THS* total hand support, *PHS* partial hand support, *WHS* without hand support, *TFS* total feet support, *PFS* partial feet support, *WFS* without feet support

**Fig. 3:**
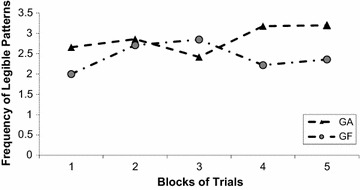
Frequency of legible patterns

The adjustable school furniture facilitates the body orientation and posture for practicing graphic skills, this should lead to a better pattern legibility. This was confirmed even though children in both groups showed changes in pattern legibility (Fig. 3). According to a Two-Way ANOVA Group (2) × Block (5) with repeated measures in the last factor, there was an interaction, F_4,147_ = 10.12, *p* < 0.001, *n*^2^ = 0.37. The conduction of a post hoc Tukey Test indicated that the two groups improved legibility with practice but GA showed more legible patterns than GF, particularly at the end of practice (last two blocks).

One should expect that adequate body posture will correlate positively with pattern legibility. The relationship between school furniture with the quality of drawing is illustrated by The Contingency Correlation Test. We found a significant correlation between some of the body posture’s components and the legibility of graphic patterns. There was a significant correlation for the GA, between total and partial feet support and pattern legibility: 0.72, *p* < 0.02. For children in the GF there was a significant correlation between total hand support and pattern legibility: 0.68, *p* < 0.03. It is worth mentioning that hand support is a component that is not dependent on the layout of the school furniture.

**Fig. 4:**
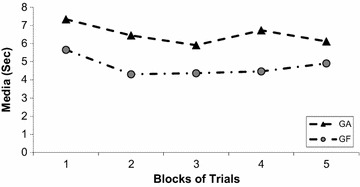
Total movement time

Pattern legibility is associated with the speed-accuracy trade-off. As the speed of execution increases, the number spatial errors might also increase. Nevertheless, it is expected that as learning progresses, the time to complete the task will decrease, hence the speed of execution will increase to a certain point, i.e. when accuracy is hindered by speed. Body posture can affect the speed-accuracy trade-off in which an adequate body posture for drawing or handwriting may allow the child to perform faster without compromising accuracy (letter legibility). The evaluation of this relationship needs to consider together the results from total time to perform the task and the spatial error. The duration to perform the task decreased for both groups during practice (Fig. 4). A Two-Way ANOVA, Group (2) × Block (5) with repeated measures in the last factor, found an interaction, F_9,345_ = 12.17, *p* < 0.002, *n*^2^ = 0.26. The conduction of a post hoc Tukey test indicated that the decrease was more marked for the GA with statistically significant differences from Block 1 to 3 and 5. For the GF the statistically significant difference was from Block 1 to 2. In spite of fewer changes during practice, the GF was faster than the GA in all blocks.

**Fig. 5:**
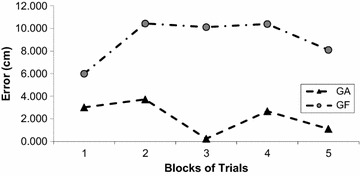
Linear spatial error

The spatial error showed different rates of change for each group with the GF showing an increase of linear spatial error and a decrease for the GA (Fig. 5). A Two Way ANOVA, Group (2) × Block (5) with repeated measures in the last factor, found an interaction, F_9,726_ = 9.17, *p* < 0.001, *n*^2^ = 0.44. The conduction of a post hoc Tukey test indicated that children in the GA showed statistically significant decrease in linear spatial error from Block 1 to Blocks 4 and 5. In another direction, children in the GF showed an increase in linear spatial error that was statistically significant from Block 1 to Blocks 2, 3, 4 and 5. The GF had also greater spatial linear error in comparison to the GA during all practice.

## Discussion

Children in both groups, with fixed (GF) and adjustable (GA) school furniture, could benefit from practicing a graphic skill. However, we found evidence that the children’s performance with the adjustable school desk was better in different ways.

First, the adjustable school furniture facilitated the adoption of body postures in particular the legibility and the accuracy with which the figure was reproduced. Children in the GA showed more legible patterns during practice and The Contingency Correlation Test confirmed this. It is true that pattern legibility improved for the children in the GF, however, as indicated by Two Way ANOVA, they showed a decrease in the number of legible patterns by the end of practice. The increase of less legible patterns may be related to the body posture the children adopted. Children in the GF rarely showed feet in contact with the surface and back support, together these two components might have contributed to a decrease in the quality of drawing. It has been shown that children who support their back in the chair and put their feet on the ground tend to write better (Amudson 2005; Feder and Majnemer 2007). In fact, one of the principal differences between the fixed and adjustable school desk school furniture the prevalence of the Total or Partial Feet Support in the body posture for children in the GA. The fact that children in the GF performed the task with more effective support for the hand on paper might be considered as a compensatory strategy to deal with the lack of precision caused by body sway due to the lack of Total Back Support in the body posture.

Second, the results in regard to duration to complete the drawing and accuracy to perform it were interesting because they corroborate the effect well known as speed-accuracy trade-off. When performing motor actions one has to balance speed and accuracy, as being too fast will hinder accuracy and vice versa (cf. Crossman and Goodeve 1983; Bootsma et al. 2004). The tests we ran for differences between the conditions (the Analysis of Variance with two factors, group and blocks) provided statistically significant interactions that support the following description: children in the GF were faster than their counterparts in the GA to perform the task during practice; however, their linear spatial error was greater along practice, hence the children in each group adopted different strategies in regard to the speed-accuracy trade-off. The Contingency Correlation Test we mentioned earlier in the discussion helps us to to suggest that this maybe influenced by the layout of the school furniture. The fixed school furniture may have contributed for the instability of shoulder and trunk leading the children to employ more hand force variation and increased speed. This combination of elements tends to disrupt coordination affecting handwriting legibility negatively (Tseng and Cermak 1993).

It is likely that the adjustable school furniture provides better conditions for the stabilization of shoulder and trunk. Children could concentrate on the task of drawing the criterion figure. The result was taking more time to complete the task, though with more accuracy. Feder and Majnemer (2007) indicated that ideal posture for the child to have better handwriting development entails sitting with feet flat on the floor and hips and low back supported against the chair back. The adjustable school desk gave exactly the opportunity for each child to adopt this posture.

## Conclusion

It is acknowledged for quite sometime that the ability to produce handwriting with fluency and legibility is important for expressing and communicating ideas (Phelps et al. 1985). In recent years there is a growing body of evidence linking handwriting and drawing skills with conceptual learning in children from a behavioral perspective (e.g. Longcamp et al. 2005) as well as neural perspective (e.g. James and Engelhardt 2012).

The appropriate conditions to facilitate children’s acquisition of handwriting and drawing skills are in order. Adjustable school furniture is important to furnish school settings with the appropriate environment for children to acquire and enhance handwriting and drawing skills much for the benefit of their overall educational development.
